# Altered activation state of circulating neutrophils in patients with neovascular age-related macular degeneration

**DOI:** 10.1186/s12979-017-0100-9

**Published:** 2017-07-27

**Authors:** Marie Krogh Nielsen, Sven Magnus Hector, Kelly Allen, Yousif Subhi, Torben Lykke Sørensen

**Affiliations:** 1 Department of Ophthalmology, Zealand University Hospital, Vestermarksvej 23, DK-4000 Roskilde, Denmark; 2 Faculty of Health and Medical Sciences, University of Copenhagen, Copenhagen, Denmark

**Keywords:** Neutrophils, Age-related macular degeneration, Choroidal neovascularization, Flow cytometry, Inflammation

## Abstract

**Background:**

Neutrophil dysfunction plays a key role in the development of diseases characterized by inflammation and angiogenesis. Here, we studied the systemic expression of neutrophil markers reflecting activation, adhesion, and resolution of inflammation in patients with neovascular age-related macular degeneration (AMD).

**Results:**

This was a prospective case-control study of patients with neovascular AMD and age-matched healthy control individuals. Patients were recruited from an outpatient program, and control individuals were recruited amongst patients’ relatives. Current smokers and individuals with either active immune-disease or ongoing cancer were not included, as these factors are known to affect neutrophil function. Fresh-drawn venous blood was processed for flow cytometric analysis of neutrophil markers. We determined percentages of positive cells and compared expression levels using fluorescence intensity measures. We found conditional differences on marker expression between patients with neovascular AMD (*n* = 29) and controls (*n* = 28): no differences were found when looking broadly, but several differences emerged when focusing on non-smokers. Here, patients with neovascular AMD had increased expression of the activity marker cluster of differentiation (CD) 66b (*P* = 0.003; Mann-Whitney U test), decreased expression of adhesion marker CD162 (*P* = 0.044; Mann-Whitney U test), and lower expression of the resolution of inflammation marker C-X-C chemokine receptor 2 (*P* = 0.044; Mann-Whitney U test).

**Conclusions:**

We present novel evidence suggesting that the activity of circulating neutrophils, sensitive to smoking, may differ in patients with neovascular AMD.

## Background

Age-related macular degeneration (AMD) is a chronic progressive disease of the aged macula [1]. In the early stages, the disease is clinically characterized by drusen, which are yellow deposits between Bruch’s membrane and the retinal pigment epithelium [1]. The late stages of AMD are characterized by localized atrophy of the retina or by choroidal neovascularizations (CNV) [1]. The latter instance is described as neovascular AMD due to its key feature where newly formed vessels of the choroid penetrate through Bruch’s membrane into the subretinal space [1]. Consequently, fluid and blood leak into the retina, irreversibly impairing vision and visual function [2]. The treatment is only able to keep vision at a stable level for some years, and neovascular AMD still remains the most common reason for irreversible vision loss in the developed world [2–4].

The pathogenesis of neovascular AMD remains incompletely understood, but ageing and dysfunction of the immune system are believed to play a key role for the disease to develop in an aged macula [5–7]. A current developing area of interest is how neutrophils play a role in disease development. This is particularly interesting since aging is the highest risk factor of developing neovascular AMD and aged neutrophils are characterized by changed surface expression and activity [5–8]. Studies have found a higher neutrophil/lymphocyte ratio in patients with neovascular AMD [9–11], and studies of donor eyes have shown infiltrating lipocalin-2-positive neutrophils at significantly higher levels in retina and the choroid in both early and late stages of AMD [12]. Lipocalin-2 is a protein expressed in neutrophils, and levels of intravitreal lipocalin-2 are significantly elevated in eyes with neovascular AMD [13].

Neutrophils are the most prominent granulocytes and are part of the innate immune system practicing granulocyte release and phagocytosis [14]. Following stimuli, the activated neutrophils adhere to the endothelial cells in the area of inflammation and migrate their way to the site of injury and infection. Neutrophilic action is mediated through an effective combination of cytotoxic granules, antimicrobial peptides, and neutrophil extracellular traps [15]. Circulating neutrophils contains myeloperoxidase (MPO), which can form a hypochlourous acid that is an efficient killer of pathogens. In Parkinson’s disease and Alzheimer’s disease, MPO is redistributed into the extracellular space where it mediates tissue damage [16, 17], which is a mechanism suspected of contributing to the pathogenesis of AMD. Accumulated MPO is a two-edged sword: it may be beneficial by clearing toxic retinal lipofuscin deposits, but may be harmful by causing lysosomal stress that results in cell death [18].

Several chemokines are suggested to play a role in recruiting monocytes/macrophages and neutrophils resulting in the formation of CNVs [19]. Zhou et al. studied laser-induced CNV on mice and found that neutrophils infiltrate the retinal tissue from the first day after stimulation and that neutrophil-depleted mice had significantly smaller CNV-response. [20] Lavalette et al. found that neutrophils infiltrated the choroid 10 h after laser stimulation expressing the proangionetic interleukin 1β [21]. Taken together, these findings support the theory that neutrophils may play an important part in the early CNV-response. In humans, we previously described that systemic levels of neutrophils correlate with CNV-lesion size in patients with neovascular AMD [22], which further supports a role for neutrophils in CNV-development in AMD.

Based on these findings, we hypothesized that systemic neutrophil expression and properties could be altered in patients with wet AMD. To investigate this further, we selected markers of interest representing key steps of neutrophil activity: activation, adhesion, and inflammation [23]. Neutrophil migration is a process, which involves activation following interaction between adhesion molecules on the neutrophils and their ligands on the endothelial cells. Cluster of differentiation (CD) 63 and CD66b are activation-molecules expressed on the surface of neutrophils after appropriate stimulation [24, 25]. CD162 (P-selectin glycoprotein ligand-1) and CD62L (P-selectin) are mediators of the first step of rolling, after which integrin molecules CD11a (lymphocyte function-associated antigen-1) and CD11b (macrophage associated antigen-1) participate [23, 24, 26]. The process of transmigration through the endothelial layer is mediated by CD54 and CD31 (platelet endothelial cell adhesion molecule-1) [27]. The degree of inflammatory activity of neutrophils can be studied by measuring the expression level of several markers. One such marker is the interleukin-1-receptor-2 (IL1-R2), which is the receptor of the highly proinflammatory cytokine interleukin-1 [28]. C-X-C chemokine receptor 2 (CXCR2) is also important in acute and chronic inflammation and is mainly regulated by interleukin-8 [29]. C-C chemokine receptor 5 (CCR5) is involved in resolution of inflammation [30]. The Duffy antigen receptor for chemokines (DARC) is suggested to play a role in inflammation since DARC seems to bind a large number of chemokines whereby it might have a protective role in preventing chemokine activation of neutrophils and inflammation [31].

In this study, we wished to study alterations of the innate immune system in neovascular AMD. We sampled blood from patients with neovascular AMD and compared them to that of aged-matched healthy control individuals. We did not include any participants who were actively smoking. Tobacco triggers acute inflammation mediated via Toll-like-receptors [32] and modulates the expression of pro-inflammatory cytokines and chemokines [33]. Also, tobacco smoking increases the risk of AMD significantly. The increase in risk is most pronounced in current smokers, but is also markedly higher in former smokers [34].

## Methods

### Study design

This was a prospective case-control study of patients with neovascular AMD and healthy controls. The study was approved by the Regional Committee of Ethics in Research in Region Zealand (SJ-142). Verbal and written informed consent was obtained from all participants prior to inclusion. The described project adhered to the tenets of the Declaration of Helsinki.

### Participants

All participants were recruited from the Department of Ophthalmology, Zealand University Hospital, Roskilde, Denmark. Patients with neovascular AMD were recruited from our retinal clinic. Healthy age-matched control individuals were relatives of the participating patients. This was an intentional strategy to better match the control group (lifestyle, diet, exposure, etc.). Since this was a hypothesis-driven study, we were unable to perform power-calculations, but based on previous experience with flow cytometric studies of systemic leukocyte markers, we aimed at recruiting at least 20 participants and stopped recruitment after successfully analyzing 29 blood samples from each group.

All participants were interviewed regarding medical history and lifestyle. Smoking was considered active if the participants had smoked at any time within the last year regardless of whether or not they had decided to stop smoking [35]. Previous smokers and non-smokers were both defined as not having smoked within the last year, and previous and non-smokers were distinguished by the latter having smoked less than 100 cigarettes (5 packs) during their entire lifetime [35]. Self-reported alcohol consumption was noted as units (=12 g ethanol) per week. We calculated body mass index using weight and height. Physical activity was assessed using a single question for epidemiological studies which, has been validated previously on patients with neovascular AMD [36, 37].

We sampled fresh venous blood from the antecubital vein in two tubes: one 5 mL ethylenediamine-tetraacetic acid coagulant containing tube for flow cytometry and one 3 mL lithium-heparin coated tube for determining C-reactive protein (CRP) level.

### Retinal diagnosis and eligibility

All participants had a comprehensive ocular examination including measurement of best-corrected visual acuity, slit-lamp examination, digital color fundus photography (Carl Zeiss, Jena, Germany), Spectral-Domain Optical Coherence Tomography, and fundus autofluorescence imaging (Spectralis HRA-OCT, SLO Heidelberg Engineering, Heidelberg, Germany). Retinal angiography using fluorescein and indocyanine green were performed where choroidal neovascularization was suspected. All retinal diagnosis was confirmed by an experienced ophthalmologist.

Healthy aged-matched controls individuals were only considered for inclusion if they had normal maculae with no more than 10 small drusen as defined in the Clinical Age-Related Maculopathy Grading System (CARMS) [38]. Any currently smoking participants were not included. Participants with any infectious diseases or immunological disorders were also not recruited, including those in immune-modulating therapy for any reason. We excluded any participant with a plasma CRP-level > 15 mg/L to avoid participants with possible ongoing infections [39]. To avoid interference with flow cytometric analyses, we did not recruit patients with neovascular AMD within 4 or 8 weeks respectively of Ranibizumab or Aflibercept therapy or immediately after retinal angiography [40].

### Flow cytometry

All samples were analyzed within 4 h of phlebotomy. We used the white blood cell count (Sysmex KX-21N™, Sysmex Corporation, Kobe, Japan) to calculate a blood volume, that would contain 5 × 10^5^ leukocytes, which we lysed in a 50 ml tube by adding red blood cell lysis buffer (Nordic Biosite AB, Täby, Sweden), and waiting 10 min in the dark at room temperature. The cells were washed three times; each time by centrifuging for 5 min at 500G, decanting the supernatant, and re-suspending in an isotonic buffer (IsoFlow Sheath Fluid, Beckman Coulter Inc., Brea, CA, USA). For each blood sample, we prepared four panels with monoclonal anti-human antibodies for cell population gating and for the markers of interest and two panels with fluorochrome-matched isotype controls: Phycoerythrin-Cyanine 7 (PC7) immunoglobulin G (IgG) 1 (Cat. No.: 400,126; BioLegend, San Diego, CA, USA), fluorescein isothiocyanate (FITC) IgG1 (Cat. No.: 400,108; BioLegend), phycoerythrin (PE) IgG1 (Cat. No.: 400,112; BioLegend), PC7 IgG2b (Cat. No.: 303,117; BioLegend), and PE IgG2b (Cat. No.: 400,212; BioLegend). We incubated samples in darkness and at room temperature, as recommended by the manufacturers. We then washed the cells, added 500 μL isotonic buffer, and re-suspended. Stained cells (*n* = 100.000) were analyzed using the flow cytometer BD FACS CANTO II (BD Biosciences, FranklinLakes, NJ, USA) and Kaluza Software (v. 1.5.20365.16139, Beckman Coulter Inc., Pasadena, CA,USA). All flow samples were analyzed using the same settings on the flow cytometer.

**Fig. 1:**
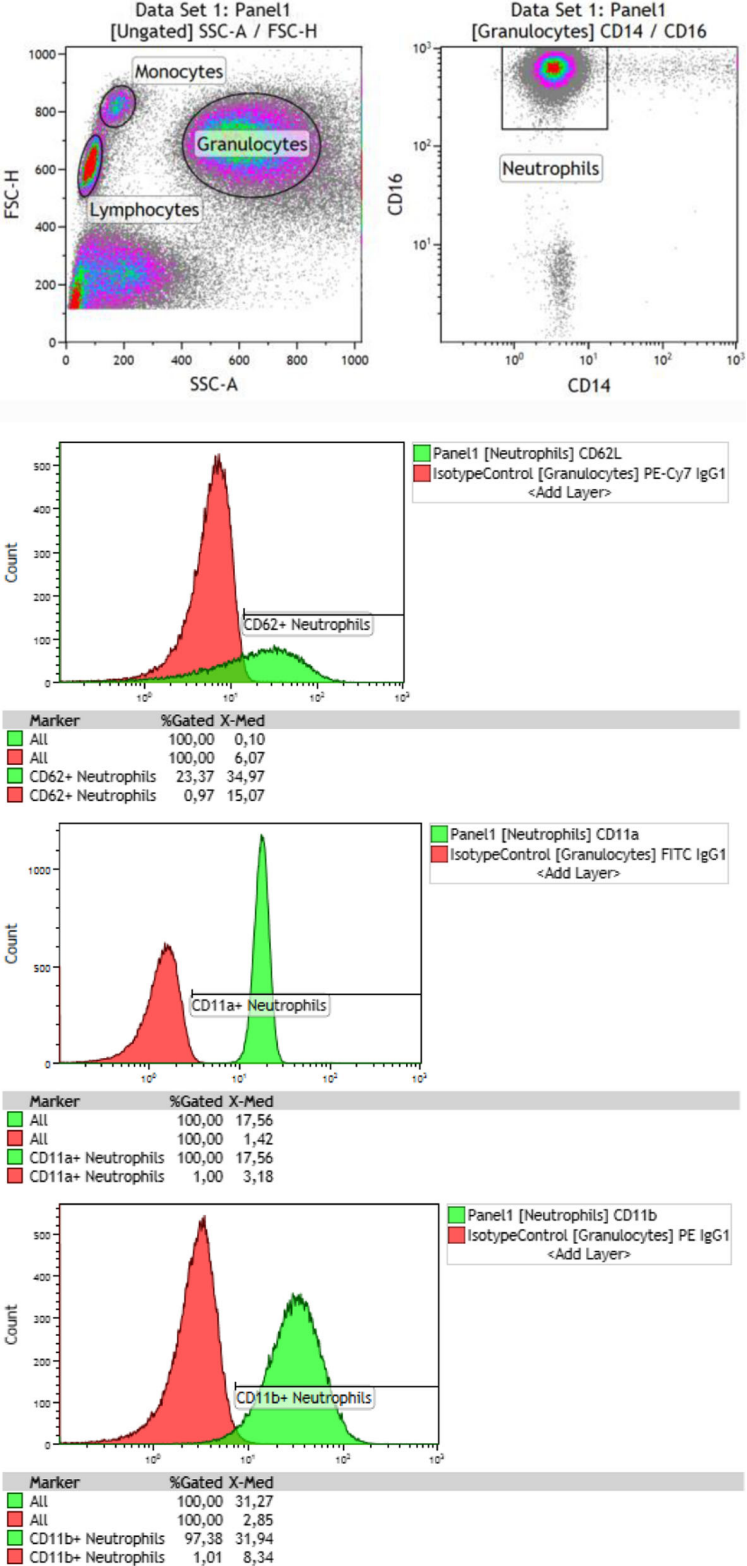
Neutrophil gating and expression analysis. Here we analyze identify CD14^dim^CD16^+^ granulocytes (neutrophils) (Top) to study percentages of CD62, CD11a, and CD11b positives and their expression level in terms of median fluorescence intensity

On a forward/side scatter plot, we isolated granulocytes, on which we used CD16 (Cat. No.: 360,712; BioLegend) and CD14 (Cat. No.: 325,616; BioLegend) for identifying neutrophils defined as CD14^dim^CD16^+^ (Fig. 1). On these neutrophils, we studied cell markers of three important functions of neutrophils: activation, adhesion, and resolution of inflammation:

- Activation: CD63 (Cat. No.: 353,009; BioLegend) and CD66b (Cat. No.: 305,103; BioLegend).
- Adhesion: CD11a (Cat. No.: 301,206; BioLegend), CD11b (Cat. No.: 301,306; BioLegend), CD31 (Cat. No.: 303,117; BioLegend), CD54 (Cat. No.: 533,107; BioLegend), CD62L (Cat. No.: 304,821; BioLegend), and CD162 (Cat. No.: 328,805; BioLegend).
- Resolution of inflammation: IL1-R2/CD121b (Cat. No: LS-C139986–100; LifeSpan BioSciences Inc., Seattle, WA, USA), CXCR2/CD182 (Cat. No.: 320,706; BioLegend), CCR5/CD195 (Cat. No.: 359,107; BioLegend), and Duffy antigen/chemokine receptor (DARC)/CD234 (Cat. No.: FAB4139P; R&D Systems Inc., Minneapolis, MN, USA).

We determined the percentage of neutrophils that were positive for the concerned marker. We gated the positive cells and defined the median fluorescence intensity (MFI).

### Data analysis and statistics

First, we compared participant characteristics between patients with neovascular AMD and the healthy control individuals (demographics, co-morbidities, lifestyle factors, and basic blood values such as white blood cell count, neutrophil percentage and count, and plasma CRP). When dealing with continuous data, we checked for normal distribution using histograms and the Kolmogorov-Smirnov test. Where normal distribution was present, data was presented using mean and standard deviation (SD) and compared using the independent samples t-test. Otherwise, data was presented using median and interquartile range (IQR) and compared using the Mann-Whitney U test. For each category (activation, adhesion, and resolution of inflammation) of markers investigated, we compared the percentage of positive neutrophils and their expression level in terms of MFI. Acknowledging the potential influence of smoking, we evaluated whether healthy controls differed in neutrophil markers between previous and non-smokers. Since this was the case, we decided to repeat all analyses on non-smokers only. All statistical analyses were made in SPSS version 23 for Mac (IBM, Armonk, NY, USA). *P*-values below 0.05 were interpret as sign of statistical significance.

## Results

We recruited a total of 61 individuals, of which 58 provided blood sample for our neutrophil study. One of the healthy control individuals had a plasma CRP >15 mg/L (22 mg/L) and was excluded from analyses. In total, 28 healthy control individuals and 29 patients with neovascular AMD were included for analyses. Mean age was 77.2 (SD: 6.6) years and 79.4 (SD: 6.1) years respectively for patients and controls (*P* = 0.192; independent samples t-test). Slightly more patients (*n* = 20, 69%) were female when compared to controls (*n* = 12, 43%) (*P* = 0.047, χ^2^-test). The patients and the control groups were generally similar in their co-morbidities and lifestyle characteristics**.** White blood cell count, and leukocyte population percentages and counts also did not differ significantly between the groups. Increased plasma CRP was more likely in patients with neovascular AMD (odds ratio 3.7, *P* = 0.033) (Table 1).

**Table 1:** Participant characteristics

	Healthy controls (*n* = 28)	Patients with neovascular AMD (*n* = 29)	*P*-value
Demographics			
Age, years, mean (SD)	77.2 (6.6)	79.4 (6.1)	0.192
Females, n (%)	12 (43)	20 (69)	0.047
Co-morbidities			
Hypertension, n (%)	13 (46)	14 (48)	0.889
Hypercholesterolemia, n (%)	9 (32)	10 (34)	0.851
Cardiovascular diseases, n (%)	12 (43)	10 (34)	0.516
Type 2 diabetes, n (%)	2 (7)	2 (7)	1.000
Lifestyle factors			
Body mass index, mean (SD)	25.8 (4.7)	26.7 (5.2)	0.506
Physically active, n (%)	6 (21)	9 (31)	0.410
Alcohol consumption, median (IQR)	7 (2 to 10)	3 (1 to 9)	0.126
Smoking status, n (%)			0.889
Previous smoker	15 (54)	15 (52)	
Non-smoker	13 (46)	14 (48)	
Blood measures			
C-reactive protein, mg/L			0.029
< 2.9 mg/L	23 (82)	16 (55)	
2.9–14.9 mg/L	5 (18)	13 (45)	
White blood cell count, 10^9^ cells/L, mean (SD)	5.9 (1.2)	6.4 (1.6)	0.212
Lymphocytes, mean (SD)			
%	29 (8)	28 (9)	0.892
10^9^ cells/L	1.7 (0.5)	1.8 (0.6)	0.654
Monocytes, mean (SD)			
%	7 (2)	6 (3)	0.457
10^9^ cells/L	0.4 (0.1)	0.4 (0.2)	0.678
Neutrophils, mean (SD)			
%	64 (8)	65 (11)	0.618
10^9^ cells/L	3.8 (1.0)	4.3 (1.5)	0.198
Neutrophils-to-lymphocytes, mean (SD)	2.52 (1.03)	2.80 (1.51)	0.422

Parametric continuous variables are presented using mean and standard deviation (SD) and tested using the independent samples t-test. Non-parametric continuous variables are presented using median and interquartile range (IQR) and tested using the Mann-Whitney U test. Categorical variables are presented using numbers (n) and percentages (%) and tested using the χ^2^-test, but due to very small numbers is co-morbidity of type 2 diabetes tested using the Fisher’s Exact test

### Activation

**Fig. 2:**
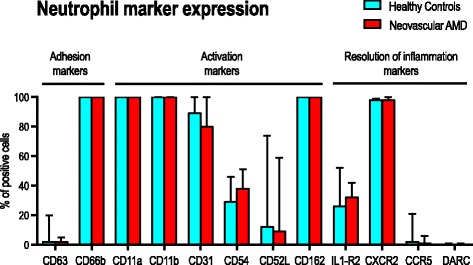
Neutrophil expression of selected markers representing key steps of neutrophil conduct: adhesion, activation, and inflammation in patients with neovascular AMD and healthy controls

**Fig. 3:**
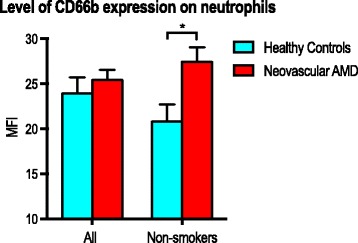
Expression of CD66b in patients with neovascular AMD and healthy controls. Analysis performed for all participants and for non-smokers

Activation markers did not differ significantly in percentage of positive neutrophils (Fig. 2). Expression level of CD63 was also similar between groups. We observed a trend towards higher expression level of CD66b in patients with neovascular AMD, but this trend did not reach a level of statistical significance. However, we repeated the analyses on non-smokers only and found that among non-smokers, expression level of CD66b is significantly higher in patients with neovascular AMD when compared to healthy controls (*P* = 0.003; Mann-Whitney U test) (Fig. 3).

### Adhesion

**Fig. 4:**
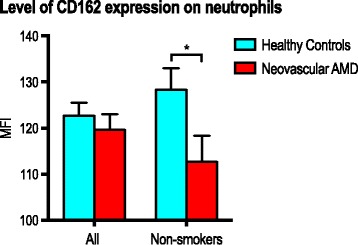
Expression of CD162 in patients with neovascular AMD and healthy controls. Analysis performed for all participants and for non-smokers

Adhesion markers did not differ significantly in percentage of positive neutrophils (Fig. 2). Expression level on the marker positive neutrophils were also similar between groups. Repeating the analyses on non-smokers only showed that CD162 expression was slightly lower in patients with neovascular AMD (*P* = 0.044; Mann-Whitney U test) (Fig. 4).

### Resolution of inflammation

**Fig. 5:**
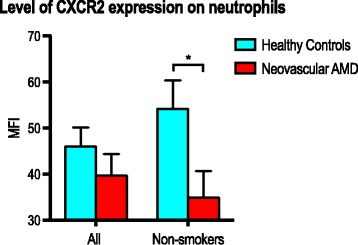
Expression of CXCR2 in patients with neovascular AMD and healthy controls. Analysis performed for all participants and for non-smokers

Resolution of inflammation markers did not differ significantly in percentage of positive neutrophils (Fig. 2). Expression level on the marker positive neutrophils were also similar for IL1-R2, CCR5, and DARC, but we observed a non-significant trend towards lower CXCR2 on neutrophils in patients with neovascular AMD. Repeating the analyses on non-smokers suggest that among non-smokers, patients with neovascular AMD have significantly lower expression of CXCR2 (*P* = 0.044; Mann-Whitney U test) (Fig. 5).

## Discussion

We find that among non-smokers, patients with neovascular AMD had increased expression of the activity marker CD66b, decreased expression of the adhesion marker CD162, and lower expression of inflammation marker CXCR2. Our results suggest that neutrophils may have specific components that play a role in neovascular AMD.

Complement activation plays a key role in the pathogenesis of AMD. Histopathological studies have found complement components in eyes with drusen [41], which are also reflected in altered levels of systemic complement markers in patients with AMD [42, 43]. Interestingly, complement activation induces CD66b overexpression and drastically reduces the neutrophils’ phagocytic capacity [25]. In light of these findings, we hypothesize that CD66b overexpression on systemic neutrophils in patients with neovascular AMD may reflect an increased systemic level of complement components that in turn inhibit the phagocytic capacity of the neutrophils. Consequently, we speculate that the drusenoid macula may lack an appropriate neutrophil response to the increased inflammatory and angiogenic drive whereby CNV formation can proceed unhampered.

Several studies suggest that AMD is associated with immunosenescence and systemic presence of low-grade inflammation [36, 44, 45]. We confirm this association in CRP, which were higher in patients with neovascular AMD. On neutrophils, such inflammatory environments influence the expression of CD162 [46, 47]. CD162 is a type 1 membrane protein that is constitutively expressed on human neutrophils and able to interact with all 3 types of selectins: P-selectin on activated platelets and endothelial cells, E-selectin on endothelial cells, and L-selectin on leukocytes [26]. This interaction between CD162 and its ligands is the first adhesion step and leads to neutrophils rolling on the endothelium prior to adhesion. Severe systemic inflammation in humans causes rapid downregulation of CD162 on neutrophils [46]. Inflammation of lesser degree have similar impact on CD162, exemplified by one study of surgical stress after cardiopulmonary bypass surgery [47]. Based on these considerations, we speculate that low-grade inflammation in patients with AMD may cause CD162 downregulation on neutrophils that in turn are less adherent.

CXCR2 is a chemokine receptor that regulates neutrophil recruitment in inflammatory contexts. Its function in inflammation and tumor-related inflammatory activities is vital and CXCR2 modulation has been suggested for treatment [48–50]. One study of CXCR2 deficient mice demonstrated that CXCR2 plays an important role for macrophage-dependent inflammatory response. In CXCR2 deficient mice, inflammatory responses were more excessive, more macrophages were recruited to sites of inflammation, and levels of anti- and pro-inflammatory cytokines were shifted towards relatively more pro-inflammatory levels [51]. Hence, CXCR2 controls the magnitude of macrophage response in inflammation. We and other groups have previously found that monocytes and macrophages may play a key role in AMD and particularly for CNV formation [22, 52–55]. Experimental laser-induced lesions on mice retinae show that macrophages are important for the CNV formation and systemic depletion of macrophages lead to significantly lower lesion size [55]. In patients with neovascular AMD, monocyte levels are increased in the first 30 days of new CNV diagnosis [22]. In light of these findings, we hypothesize that a lower expression of CXCR2 and the aged and drusenoid macula may be an ill-matched couple that orchestrates a more excessive macrophage activity and pro-inflammatory environment where CNV formation can occur. Interestingly, rheumatoid arthritis which is associated with CXCR2 deficiency is also associated with developing AMD later in life [56, 57].

Limitations of this study should be noted when interpreting its results. Importantly, this was an observational, case-control study, which can only associate but not infer on causality. Thus, we cannot exclude that these findings may also reflect a post-CNV state in the blood. We can only speculate on causality. However, findings of previous studies suggesting that complement dysfunction, low-grade inflammation, and CXCR2 deficiency all comes prior to onset of AMD gives reasons to expect causality [45, 57, 58]. The exploratory approach in the study and the limited group sizes did not permit meaningful stratifications based on single nucleotide polymorphisms that could be interesting, e.g. in the complement system [58, 59] in light of our findings on CD66b. Our study design cannot determine whether the findings reflect immunological dysfunction that are seen in a broad range of retinal diseases or mechanisms that specifically lead to AMD. Future studies need to investigate other retinal and ophthalmological diseases to clarify such aspects.

## Conclusions

In summary, we find that in non-smokers, patients with neovascular AMD present with neutrophils that have increased expression of the activity marker CD66b, decreased expression of the adhesion marker CD162, and lower expression of the resolution of inflammation marker CXCR2. Circulating neutrophils may play a role for neovascular AMD and experimental studies are warranted to fully clarify how and when these neutrophil dysfunctions contribute to disease development.

## Abbrevations

AMD: Age-related macular degeneration
CNV: Choroidal neovascularization
CD: Cluster of differentiation
PC7: Phycoerythrin-Cyanine 7
IgG: Immunoglobulin G
FITC: Fluorescein isothiocyanate
PE: Phycoerythrin
CXCR2: C-X-C chemokine receptor 2
CCR5: C-C chemokine receptor 5
DARC: Duffy antigen receptor for chemokines
CARMS: Clinical Age-Related Maculopathy Grading System
CRP: C-reactive protein
MFI: Median fluorescence intensity
SD: Standard deviation
IQR: Interquartile range
